# Supplementary material for: Stallion Sperm Freezing with Different Extenders: Role of Antioxidant Activity and Nitric Oxide Production
Source: Animals (Basel). 2024 Aug 25;14(17):2465. doi: 10.3390/ani14172465 (PMC11394550; doi:10.3390/ani14172465)
Supplement: Supplementary file 1 [file animals-14-02465-s001.zip › animals-3155373-supplementary.pdf]

Table S1. Sperm kinetics, mitochondrial activity (MMP), and H_2O_2 content (ROS) in horse semen diluted (1:2) with three shipping/centrifugation extenders (SCEs) and submitted to a slow temperature decrease (from 38 to 20 °C, i.e., 0.10- 0.15 °C min^{-1}).

Stallion	SCEs	TotMot %	PROG %	VCL µm/s	VSL µm/s	VAP µm/s	MMP F_0B/F_0A	ROS a.u.	ABTS µM	FRAP µM	NOx µM
#1	1	77.7 ± 0.0	25.3 ± 0.7	63.4 ± 1.4	26.0 ± 3.4	38.0 ± 2.6	35.3 ± 0.7	28.8 ± 6.3	384 ± 8	512 ± 19	n.d.
	2	97.1 ± 0.3	29.9 ± 0.6	67.1 ± 0.2	30.2 ± 1.1	44.2 ± 1.7	9.7 ± 0.9	29.5 ± 1.1	108 ± 10	272 ± 13	n.d.
	3	93.1 ± 0.3	31.7 ± 3.1	63.6 ± 1.4	29.5 ± 0.5	41.3 ± 1.2	23.0 ± 1.6	19.9 ± 0.4	137 ± 8	467 ± 9	n.d.
#2	1	81.6 ± 4.1	32.7 ± 9.5	62.1 ± 2.5	26.0 ± 1.2	41.7 ± 0.9	47.5 ± 0.4	20.5 ± 0.7	570 ± 6	625 ± 30	n.d.
	2	84.9 ± 5.1	19.0 ± 1.2	66.1 ± 4.3	24.5 ± 0.2	49.9 ± 2.0	35.9 ± 4.9	18.5 ± 0.2	552 ± 4	506 ± 20	n.d.
	3	97.4 ± 0.2	23.2 ± 0.9	60.9 ± 3.2	20.7 ± 0.7	36.5 ± 1.4	17.1 ± 2.2	21.6 ± 1.1	625 ± 9	480 ± 30	n.d.
#3	1	72.2 ± 4.2	26.6 ± 0.2	104.8 ± 5.0	59.2 ± 3.1	82.8 ± 7.3	34.1 ± 1.9	26.6 ± 0.4	327 ± 27	307 ± 10	n.d.
	2	68.3 ± 1.4	17.0 ± 2.6	102.4 ± 3.5	55.1 ± 5.5	82.9 ± 5.8	47.8 ± 4.1	27.6 ± 3.3	1153 ± 9	544 ± 33	n.d.
	3	76.9 ± 6.5	28.5 ± 1.5	92.3 ± 1.6	50.5 ± 4.1	69.3 ± 5.5	33.6 ± 0.4	25.6 ± 0.7	324 ± 11	459 ± 28	n.d.
#4	1	67.5 ± 6.4	21.4 ± 2.6	107.3 ± 8.9	36.8 ± 4.3	59.7 ± 4.7	38.4 ± 2.1	29.5 ± 0.7	n.d.	339 ± 17	n.d.
	2	52.1 ± 4.4	10.2 ± 3.8	110.8 ± 5.2	34.9 ± 0.8	65.8 ± 1.2	34.5 ± 0.7	30.5 ± 2.6	202 ± 6	204 ± 7	n.d.
	3	56.0 ± 4.3	19.4 ± 2.7	96.2 ± 0.2	37.9 ± 1.4	63.8 ± 5.9	27.0 ± 5.8	27.7 ± 4.2	n.d.	289 ± 16	304 ± 10
#5	1	75.3 ± 0.2	14.7 ± 0.3	106.4 ± 8.2	33.6 ± 0.8	65.3 ± 3.7	21.9 ± 4.7	29.7 ± 2.1	766 ± 10	262 ± 18	72 ± 5
	2	74.6 ± 3.6	12.2 ± 2.6	118.9 ± 1.6	34.5 ± 3.2	69.9 ± 5.5	8.0 ± 1.5	25.2 ± 3.5	325 ± 14	252 ± 12	n.d.
	3	62.4 ± 11.3	14.1 ± 2.1	103.4 ± 1.4	32.5 ± 2.7	61.5 ± 6.2	14.8 ± 3.0	23.5 ± 0.8	543 ± 5	457 ± 26	n.d.
#6	1	82.7 ± 1.4	18.0 ± 2.1	84.0 ± 0.0	29.2 ± 0.1	50.4 ± 1.1	37.3 ± 1.3	21.1 ± 0.4	37 ± 13	258 ± 16	n.d.
	2	87.0 ± 6.7	14.3 ± 3.5	75.1 ± 5.2	25.7 ± 2.3	41.9 ± 4.1	12.0 ± 0.4	23.5 ± 0.1	257 ± 5	197 ± 12	n.d.
	3	97.7 ± 1.5	22.3 ± 2.3	106.3 ± 6.1	33.6 ± 1.3	59.4 ± 4.4	19.4 ± 1.5	24.7 ± 1.7	120 ± 3	337 ± 17	n.d.
#7	1	79.9 ± 0.5	21.8 ± 6.8	84.2 ± 7.5	29.6 ± 1.2	52.5 ± 2.9	35.6 ± 3.2	23.8 ± 0.4	346 ± 14	329 ± 31	n.d.
	2	82.2 ± 1.7	15.2 ± 1.6	86.7 ± 3.7	28.2 ± 1.8	53.9 ± 3.9	18.6 ± 2.0	22.4 ± 1.2	551 ± 7	373 ± 33	n.d.
	3	90.8 ± 1.5	26.8 ± 1.3	100.9 ± 2.5	41.2 ± 4.4	69.8 ± 7.6	15.1 ± 2.4	21.1 ± 0.3	387 ± 6	316 ± 10	n.d.
#8	1	48.4 ± 4.8	15.9 ± 1.0	104.5 ± 2.9	35.6 ± 2.8	60.7 ± 8.4	5.8 ± 1.9	22.8 ± 5.0	592 ± 6	378 ± 23	27 ± 6
	2	57.3 ± 1.1	18.9 ± 2.4	83.4 ± 21.1	33.9 ± 7.9	54.5 ± 16.1	6.4 ± 0.1	21.3 ± 0.3	455 ± 5	317 ± 13	n.d.
	3	71.9 ± 1.8	23.2 ± 0.4	90.7 ± 1.5	33.9 ± 2.2	56.5 ± 4.5	6.5 ± 0.3	24.7 ± 1.1	346 ± 3	678 ± 22	27 ± 7

#9	1	99.8 ± 0.0	19.4 ± 0.9	131.5 ± 38.2	36.8 ± 10.5	67.7 ± 19.7	35.4 ± 1.3	19.7 ± 1.0	798 ± 28	479 ± 45	121 ± 10
	2	98.5 ± 0.2	24.7 ± 1.4	113.5 ± 5.7	38.4 ± 3.2	67.3 ± 4.4	14.0 ± 0.3	18.1 ± 0.8	907 ± 8	699 ± 48	95 ± 7
	3	99.0 ± 0.1	30.5 ± 6.4	118.0 ± 0.9	41.6 ± 1.2	70.6 ± 0.3	9.1 ± 0.8	19.9 ± 1.4	941 ± 8	507 ± 19	69 ± 4
#10	1	96.9 ± 0.2	34.6 ± 1.2	108.5 ± 5.0	45.3 ± 2.2	74.5 ± 4.2	58.1 ± 0.1	20.7 ± 0.3	n.d.	261 ± 16	n.d.
	2	97.6 ± 0.3	33.9 ± 3.0	106.7 ± 3.4	43.6 ± 1.2	72.1 ± 0.2	68.7 ± 1.0	24.2 ± 0.1	31 ± 6	267 ± 13	n.d.
	3	97.7 ± 1.7	24.4 ± 1.8	109.1 ± 5.3	39.9 ± 2.9	69.7 ± 6.1	82.6 ± 0.0	24.1 ± 1.1	1416 ± 13	338 ± 6	n.d.

Shipping and centrifugation extenders: (1) Equiplus, (2) Botusemen, and (3) INRA 96; fluorescence intensity ratio of the JC-1 emission peaks (F_0B/F_0A); arbitrary units (a.u.); not detectable (n.d.).

Table S2. Sperm kinetics, mitochondrial activity, and reactive oxygen species (ROS) content in frozen/thawed horse semen diluted with four freezing extenders.

**Stallion**	**Extender**	**TotMot** %	**PROG** %	**VCL** µm/s	**VSL** µm/s	**VAP** µm/s	**MMP** F_0B/F_0A	**ROS** a.u.	**ABTS** µM	**FRAP** µM	**NOx** µM
#1	1	29.4 ± 2.8	7.6 ± 0.5	52.0 ± 0.7	24.2 ± 1.8	32.4 ± 1.8	25.4 ± 2.9	11.2 ± 1.8	89 ± 6	743 ± 60	125 ± 13
	2	35.7 ± 0.6	9.5 ± 2.6	51.0 ± 2.0	26.0 ± 0.9	33.0 ± 1.0	18.9 ± 6.4	7.3 ± 0.2	458 ± 49	628 ± 39	n.d
	3	34.6 ± 0.5	5.0 ± 1.5	48.1 ± 8.1	19.6 ± 3.5	29.0 ± 5.7	20.8 ± 1.2	4.5 ± 0.4	646 ± 71	344 ± 18	n.d
	4	28.9 ± 11.2	5.3 ± 3.1	48.1 ± 3.8	20.9 ± 0.0	28.7 ± 1.1	15.6 ± 1.0	3.7 ± 1.6	n.d	554 ± 50	n.d
#2	1	29.7 ± 2.4	14.3 ± 3.7	59.0 ± 5.1	29.5 ± 5.6	37.1 ± 7.1	13.5 ± 1.8	2.7 ± 0.3	127 ± 11	914 ± 58	188 ± 15
	2	29.5 ± 5.7	9.9 ± 1.4	57.4 ± 2.5	22.7 ± 1.5	31.5 ± 1.1	23.4 ± 4.0	2.5 ± 0.1	334 ± 15	703 ± 31	72 ± 3
	3	23.5 ± 0.8	3.4 ± 0.1	53.0 ± 2.6	18.7 ± 1.5	28.3 ± 0.8	11.3 ± 0.7	2.8 ± 0.5	1232 ± 160	507 ± 27	n.d
	4	9.9 ± 0.3	2.0 ± 1.1	50.8 ± 4.6	20.9 ± 3.6	29.8 ± 5.3	16.8 ± 0.5	2.0 ± 0.0	n.d	835 ± 50	n.d
#3	1	30.7 ± 3.9	19.7 ± 0.7	76.5 ± 12.4	46.4 ± 11.1	57.7 ± 11.6	2.5 ± 0.3	17.3 ± 2.4	n.d	964 ± 64	498 ± 28

	2	37.2 ± 1.4	22.9 ± 0.9	79.6 ± 10.9	49.1 ± 13.3	57.8 ± 15.9	3.1 ± 0.5	11.6 ± 0.2	n.d	951 ± 55	151 ± 16
	3	29.4 ± 0.1	10.9 ± 0.1	58.2 ± 8.4	33.3 ± 8.5	42.1 ± 9.4	2.7 ± 0.1	13.5 ± 0.8	949 ± 65	377 ± 20	n.d
	4	23.4 ± 3.3	14.0 ± 6.3	67.9 ± 7.7	37.2 ± 2.2	45.7 ± 2.5	4.5 ± 1.2	11.6 ± 0.0	n.d	862 ± 65	n.d
#4	1	11.4 ± 1.5	5.4 ± 0.5	73.7 ± 13.3	35.4 ± 3.9	49.4 ± 8.9	2.4 ± 0.1	11.8 ± 0.2	n.d	1012 ± 50	n.d
	2	9.4 ± 2.9	3.6 ± 2.6	67.2 ± 12.9	29.8 ± 10.2	41.2 ± 13.2	4.7 ± 0.5	13.8 ± 0.9	n.d	846 ± 34	300 ± 26
	3	9.3 ± 0.6	4.0 ± 1.4	67.4 ± 0.2	30.4 ± 7.4	41.6 ± 8.4	2.3 ± 0.1	11.4 ± 0.2	1099 ± 87	381 ± 21	n.d
	4	8.1 ± 0.4	3.9 ± 0.4	70.1 ± 3.6	36.3 ± 8.4	46.5 ± 11.8	1.3 ± 0.2	12.0 ± 0.7	n.d	665 ± 28	n.d
#5	1	12.9 ± 0.5	3.2 ± 0.3	60.4 ± 2.1	23.7 ± 1.7	34.7 ± 0.7	5.1 ± 0.1	16.1 ± 1.3	n.d	1217 ± 70	n.d
	2	11.6 ± 2.6	4.6 ± 0.2	69.7 ± 5.2	33.6 ± 0.6	45.7 ± 0.9	5.1 ± 1.2	18.0 ± 0.2	n.d	907 ± 34	117 ± 7
	3	24.9 ± 4.1	9.2 ± 0.1	66.2 ± 9.7	29.1 ± 7.9	40.2 ± 11.2	17.0 ± 1.1	20.2 ± 0.3	701 ± 80	407 ± 16	n.d
	4	18.3 ± 1.0	5.9 ± 0.9	63.5 ± 3.3	26.7 ± 5.4	38.1 ± 6.2	13.6 ± 0.3	27.1 ± 3.4	n.d	723 ± 46	n.d
#6	1	23.3 ± 3.2	2.8 ± 0.1	53.0 ± 0.1	19.3 ± 0.2	30.5 ± 0.8	10.7 ± 0.9	5.8 ± 0.7	n.d	848 ± 50	27 ± 4
	2	19.8 ± 1.1	2.9 ± 0.0	52.3 ± 8.7	20.3 ± 5.1	31.2 ± 7.3	11.8 ± 2.4	7.9 ± 0.6	n.d	350 ± 13	31 ± 2
	3	50.9 ± 3.0	5.9 ± 1.4	56.9 ± 1.5	19.2 ± 0.5	31.7 ± 0.3	12.1 ± 3.3	8.7 ± 0.6	421 ± 28	274 ± 10	n.d
	4	25.0 ± 1.0	6.3 ± 0.8	62.4 ± 0.1	24.3 ± 1.0	34.9 ± 0.7	6.4 ± 0.8	4.9 ± 0.4	n.d	727 ± 35	n.d
#7	1	25.8 ± 0.6	17.7 ± 1.9	70.1 ± 4.3	41.0 ± 1.0	50.8 ± 0.3	7.9 ± 0.9	8.6 ± 0.1	n.d	1095 ± 80	27 ± 2
	2	28.4 ± 6.1	8.8 ± 1.1	58.8 ± 10.3	26.6 ± 9.6	36.6 ± 11.6	7.9 ± 0.4	14.1 ± 0.2	n.d	718 ± 21	231 ± 26
	3	43.6 ± 7.1	20.4 ± 2.5	64.8 ± 4.6	27.9 ± 1.8	37.5 ± 1.2	6.0 ± 0.3	11.5 ± 2.0	578 ± 65	267 ± 10	n.d
	4	22.2 ± 3.5	14.3 ± 1.6	68.3 ± 12.3	35.3 ± 11.6	44.0 ± 14.0	11.8 ± 0.2	12.2 ± 0.0	n.d	681 ± 37	n.d
#8	1	15.8 ± 1.1	9.8 ± 0.9	76.1 ± 7.5	50.5 ± 8.8	59.0 ± 10.4	10.6 ± 0.4	15.8 ± 1.3	n.d	902 ± 33	n.d
	2	10.8 ± 1.2	4.8 ± 0.8	56.1 ± 1.0	35.4 ± 5.8	38.7 ± 4.1	12.9 ± 0.6	17.9 ± 0.6	n.d	760 ± 26	155 ± 8
	3	13.6 ± 4.3	6.8 ± 2.4	58.4 ± 6.3	34.6 ± 10.6	40.3 ± 9.9	13.8 ± 0.6	20.7 ± 0.4	n.d	379 ± 12	n.d
	4	20.9 ± 2.4	4.5 ± 1.9	50.8 ± 1.6	24.5 ± 0.3	33.4 ± 0.2	7.9 ± 0.7	26.3 ± 5.4	n.d	819 ± 50	n.d
#9	1	46.2 ± 4.8	18.8 ± 1.2	72.2 ± 15.5	47.5 ± 15.3	56.3 ± 16.5	8.6 ± 0.1	16.8 ± 1.2	n.d	896 ± 30	27 ± 5
	2	25.1 ± 0.8	13.1 ± 0.2	73.7 ± 3.1	54.0 ± 0.4	58.0 ± 0.1	10.3 ± 2.0	11.5 ± 0.7	n.d	996 ± 49	61 ± 2
	3	35.9 ± 2.0	5.5 ± 1.3	49.2 ± 5.6	21.5 ± 2.5	31.2 ± 4.1	5.9 ± 0.8	15.3 ± 1.0	359 ± 23	350 ± 18	38 ± 5
	4	60.2 ± 7.0	4.0 ± 0.8	51.3 ± 3.2	18.5 ± 0.6	30.8 ± 1.3	12.5 ± 0.7	16.4 ± 0.3	n.d	551 ± 20	n.d
#10	1	59.0 ± 9.5	18.3 ± 1.6	62.1 ± 3.6	26.6 ± 1.3	38.7 ± 0.9	2.8 ± 0.5	20.6 ± 2.8	n.d	712 ± 30	72 ± 8

2	51.9 ± 10.7	15.4 ± 1.6	61.9 ± 2.8	24.7 ± 1.1	35.8 ± 0.3	2.8 ± 0.5	27.9 ± 3.0	85 ± 7	422 ± 24	n.d
3	38.9 ± 11.5	14.8 ± 1.1	63.0 ± 3.4	29.6 ± 7.6	39.5 ± 6.2	2.6 ± 0.3	22.6 ± 0.9	606 ± 48	589 ± 31	n.d
4	38.3 ± 4.7	15.4 ± 0.6	61.3 ± 11.9	26.2 ± 6.3	35.7 ± 7.8	1.3 ± 0.0	20.2 ± 0.9	n.d	626 ± 39	n.d

Freezing extenders: (1) Spectrum Dual Red, (2) BotuCrio, (3) INRAFreeze, and (4) HF-20; fluorescence intensity ratio of the JC-1 emission peaks (F_0B/F_0A); arbitrary units (a.u.); not detectable (n.d.).

Highlights:

Semen extenders vary in antioxidant capacity and nitric oxide content levels.

Stallion sperm freezing tolerance depends on the type of freezing extenders used.

FRAP antioxidant capacity in seminal plasma isn't tied to cooling and freezing sperm functions.

ABTS antioxidant capacity in seminal plasma relates to kinetics of cooled sperm.

Nitric oxide content in seminal plasma relates to certain functions of cooled sperm.
